# Design of a Large-Scale Piezoelectric Transducer Network Layer and Its Reliability Verification for Space Structures

**DOI:** 10.3390/s20154344

**Published:** 2020-08-04

**Authors:** Yuanqiang Ren, Jingya Tao, Zhaopeng Xue

**Affiliations:** Research Center of Structural Health Monitoring and Prognosis, State Key Lab of Mechanics and Control of Mechanical Structures, Nanjing University of Aeronautics and Astronautics, Nanjing 210016, China; tjy@nuaa.edu.cn (J.T.); xuezhaopeng@nuaa.edu.cn (Z.X.)

**Keywords:** space structures, structural health monitoring, large-scale lightweight PZT network, extreme environment, reliability verification

## Abstract

As an effective structural health monitoring (SHM) technology, the piezoelectric transducer (PZT) and guided wave-based monitoring methods have attracted growing interest in the space field. When facing the large-scale monitoring requirements of space structures, a lot of PZTs are needed and may cause problems regarding to additional weight of connection cables, placement efficiency and performance consistency. The PZT layer is a promising solution against these problems. However, the current PZT layers still face challenges from large-scale lightweight monitoring and the lack of reliability assessment under extreme space service conditions. In this paper, a large-scale PZT network layer (LPNL) design method is proposed to overcome these challenges, by adopting a large-scale lightweight PZT network design method and network splitting–recombination based integration strategy. The developed LPNL offers the advantages of being large size, lightweight, ultra-thin, flexible, customized in shape and highly reliable. A series of extreme environmental tests are performed to verify the reliability of the developed LPNL under space service environment, including extreme temperature conditions, vibration at different flying phases, landing impact, and flying overload. Results show that the developed LPNL can withstand these harsh environmental conditions and presents high reliability and functionality.

## 1. Introduction

Space vehicles, such as reusable launch vehicles, have become critical to many sectors of national defense and civilian-oriented markets in recent decades [1,2]. Due to the use of new materials, such as composites and exposure to extreme environmental conditions, it is inevitable that reliable inspection methods are required for safety control and the maintenance of space structures to avoid potential catastrophic failure [3,4]. Structural health monitoring (SHM) has proved to be an effective technology in increasing the reliability of structures and reducing maintenance cost and is particularly attractive in many application fields [5,6,7,8,9]. As a matter of fact, SHM is already in the forefront of space applications—various SHM methods have been proposed by adopting different kinds of sensors, including the piezoelectric transducer (PZT) [10], fiber optic sensors [11,12], acoustic emission sensors [13] and micro-electromechanical systems (MEMS) [14].

Among the existing methods, the PZT and guided wave (GW)-based monitoring methods, which have the advantages of long propagation distance, being sensitive to small defect, applicable for both metallic structures and composite structures, is considered as one of the most promising SHM technologies [15,16,17,18]. Many researchers have been interested in adopting this method to perform structural damage monitoring research in the space field [19,20,21,22]. For example, Yang et al. [23] developed a PZT-embedded sensor washer based SHM system to autonomously detect the mechanical integrity of the standoff carbon-carbon (C-C) thermal protection system panels. Kundu et al. [24] studied the applicability of monitoring the point of impact and detecting delamination in the thermal protection system based on guided waves. Zagrai et al. [25] presented a discussion of factors affecting realization of the piezoelectric active sensors based SHM, which focused on improperly tightened bolts, assessment of adhesive bonds, and embedded material characterization. Toader et al. [26] adopted piezoelectric wafer active sensors and neural network to perform mechanical damage diagnosis of spacecraft structures.

It can be seen from the current research that the adopted PZTs are manually placed on structure one by one, which causes challenges to the reliable implementation of the method. On one hand, it is time-consuming and lack of consistency, especially when a lot of PZTs are needed to work as a network to realize the health monitoring of large-scale space structures. On the other hand, each PZT needs an individual cable to receive excitation signal or transmit GW signal. Therefore, there will be a considerable additional weight of the cables when numbers of PZTs are adopted, which is usually unacceptable [27,28]. Besides, the placement of cables is also a challenge for onboard SHM applications, considering the complex structural forms and closed regions.

The challenges mentioned above have given risen to the development of PZT layer design methods. Towards practical engineering applications, Lin and Chang et al. [29] proposed a concept called SMART layer by placing piezoelectric sensors in a flexible interlayer, which offers the benefits of reducing additional weight and enhancing placement efficiency. Qiu et al. [30] designed a kind of piezoelectric transducer layer with electromagnetic shielding and high connection reliability, which is verified by experiments on noise and crosstalk suppression, temperature durability and strength fatigue durability. By using inkjet printing technology to print conductive network, Bekas et al. [31] developed an innovative diagnostic film and assessed its reliability through extensive tests simulating the operational environment of an aircraft. Additionally, Salmanpour et al. [32] investigated and compared the robustness of several kinds of current PZT layers under aeronautic operational environment, according to the existing DO-160 test methods.

Although theses developed layers are promising for onboard SHM, two main issues still exist when facing real space applications. The first issue is that the size of the proposed layers is relatively small and the number of PZTs that these layers contain is limited. When performing the large-scale monitoring of space structures, multiple layers should be adopted to work together, which will cause problems regarding to additional weight, placement efficiency and consistency. Therefore, an integrated and lightweight network, which can contain lots of PZTs, is needed. The second issue is related to the reliability under service environment. Since space structures may subject to harsh environmental conditions, such as extreme high and low temperature, thermal shock, vibration, landing impact, overload, and so on, the network’s reliability must be tested for these conditions. However, the current environmental tests on PZT layers are basically performed under normal aeronautical service environments. Environmental verifications for space service conditions, which are much harsher, are necessary and should be considered.

Aiming at the two issues, this paper proposes a large-scale PZT network layer (LPNL) development method based on flexible printed circuit (FPC) technology. The LPNL can contain as many PZTs as the monitoring strategy requires without any additional connection cables, and has the characteristics of being large size, lightweight, ultra-thin, flexible, customized in shape and highly reliable. These characteristics enable the efficient, accurate and consistent PZT network placement, and make the developed LPNL suitable for onboard space SHM applications. A serial of tests under extreme environmental conditions, which are specific to the service environment of space structures, are carried out and verify the reliability of the developed LPNL.

The rest of this paper is organized as follows. Section 2 introduces the design method of the LPNL. Section 3 presents the reliability verification of the developed LPNL under a series of extreme environmental conditions, and further verifies its damage diagnostic performance after all the environmental tests. Section 4 gives conclusions.

## 2. Development of the Large-Scale PZT Network Layer

The proposed development method mainly contains two aspects, namely the large-scale lightweight network design method and the network splitting–recombination method. The former focuses on eliminating the dependence on traditional PZT cables and realizing the lightweight design of a large-scale PZT network. The latter aims at overcoming the current manufacturing limitation on network’s dimension and obtaining the reliable integration of LPNL.

### 2.1. Large-Scale Lightweight Network Design Method

The large-scale lightweight network design method is realized based on FPC technology, which provides an effective strategy of packaging the traditional wired PZT network into a flexible and customizable polyimide film. This strategy offers the benefits of accurately arranging the position of every PZT in the network, replacing the traditional connection cables with printed circuits to greatly reduce the additional weight and being suitable for complex structural forms. Additionally, the placement of the PZT network on structures can be completed at one time, which is helpful to improve the efficiency and keep the consistency. The main design process is given as follows.

**Figure 1:**
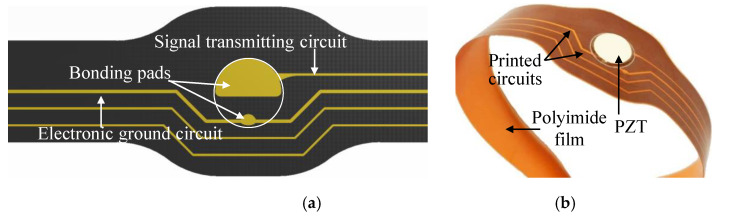
A small section of a developed LPNL. (**a**) The designed FPC. (**b**) The manufactured layer with PZT.

(1) Localization of PZTs: According to the monitoring requirements, the number of PZTs and their positions on the structure are known. Therefore, when designing the network’s FPC, every PZT can be exactly located by setting bonding pads at corresponding position. As shown in Figure 1a, the bonding pads are used to connect PZT and the polyimide film by soldering. This is also able to ensure the accuracy of distance between adjacent PZTs. In this work, PZT-5A is adopted as a PZT sensor, which has a diameter of 8 mm and a thickness of 0.5 mm and can work within the temperature range from –55 °C to 200 °C.

(2) Customizable shape of network: The shape of the large-scale lightweight network depends on the polyimide film, which is customizable through FPC and is the network’s flexible base, as shown in Figure 1. Based on the localization of PZTs, the customizable shape design of the network can be realized through designing the film according to the form of the structure. The thickness of the adopted polyimide film is only 0.2 mm, making it able to pass through the bottom of complex structural form such as beams and stiffeners. Hence, the developed LPNL can be easily deployed on space structures and will not affect the assembly of structures. Besides, the adopted polyimide has good temperature adaptability.

(3) Distribution of printed signal circuits: After finishing the network’s shape design, the distribution of printed signal circuits is planned to replace the traditional cables to transmit GW signals of all the PZTs. For each PZT, one signal transmitting circuit and one electronic ground circuit are needed, as shown in Figure 1. The signal transmitting circuit is connected to the positive pole of the PZT, and the electronic ground circuit is connected to the ground. It should be noted that the latter can be shared by multiple PZTs to reduce the total area of the network’s printed circuits. In order to reduce crosstalk between adjacent signal transmitting circuits, their distance should be at least two-times larger than the width of circuit [33].

### 2.2. Network Splitting-Recombination Method

**Figure 2:**
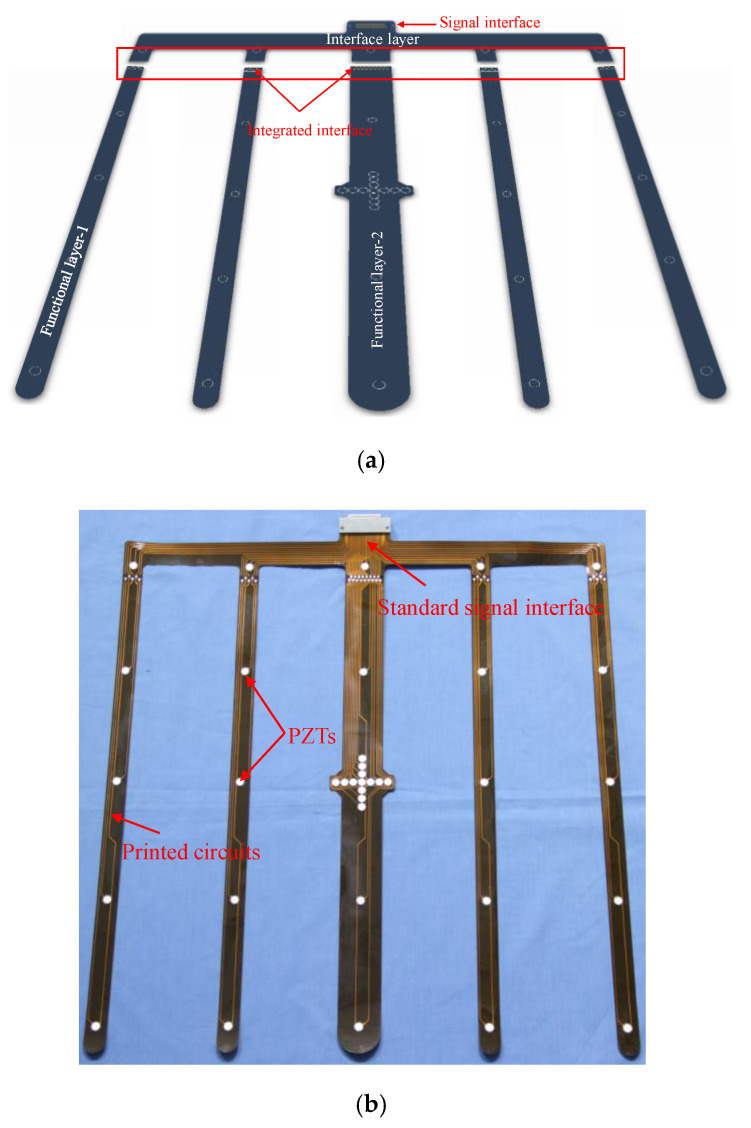
Example of a developed LPNL. (**a**) Schematic diagram of the recombination process. (**b**) The manufactured LPNL.

As for the manufacture of LPNL, a network splitting–recombination method is proposed to overcome the current limitation of FPC process on dimension. A LPNL is took as an example to be analyzed here, as shown in Figure 2. The LPNL has a dimension of 565 mm × 500 mm and can connect a total of 37 PZTs, according to the monitoring requirement of a stiffened composite structure. As for this LPNL, it is split into one interface layer and two kinds of functional layers, namely functional layer-1 and functional layer-2, in the network splitting step, due to the exceeded dimension. In the recombination step, all the functional layers are connected to the interface layer by soldering and conducting resin through the reserved integrated interface on both the functional layer and interface layer. Figure 2a gives the schematic diagram of the recombination process and the connection parts of different layers. Figure 2b shows the back side of the manufactured LPNL, which contains 37 PZTs, printed circuits and a standard signal interface.

### 2.3. Main Characteristics of The Developed LPNL

Based on the proposed methods, the LPNL can be realized and has the following characteristics: (1) Large size—the dimension is no longer limited by the current manufacturing processes of FPC; therefore, the number of PZTs in the network is also not limited; (2) Lightweight—the additional weight is about 55 g/m^2^, which is reduced by more than 80% compared with the traditional PZT network with cables; (3) Ultra-thin—the thickness of the area with PZT is about 0.8 mm, the other areas is about 0.3 mm; (4) Flexibility—the LPNL has good flexibility and can effectively fit complex structural surfaces; (5) Customized shape—the shape is customizable, making it suitable for complex structures and will not affect the assembly of structures; (6) High reliability—the developed LPNL can withstand extreme space service environment, which will be verified in Section 3.

Besides, by adopting specific curing methods, the developed LPNLs can be either surface-mounted on metal and composite structures or embedded in the composite structures.

## 3. Reliability Verification under Extreme Environmental Conditions

In order to verify the reliability under the extreme service environment of space structures, a developed LPNL is surface mounted on a carbon fiber composite plate and adopted to perform environmental tests. It should be noted that the test profiles all come from real space applications and are introduced as follows. After finishing all the environmental tests, a damage diagnostic test is further carried out to assess the LPNL’s functionality.

There are four kinds of environmental tests: (1) Temperature environment, including high/low temperature and thermal shock; (2) High vibration environment, including two different high frequency random vibration conditions; (3) Impact environment, including an impact response spectrum and a half sinusoid spectrum; (4) Overload environment, including three different directions of high overload.

**Figure 3:**
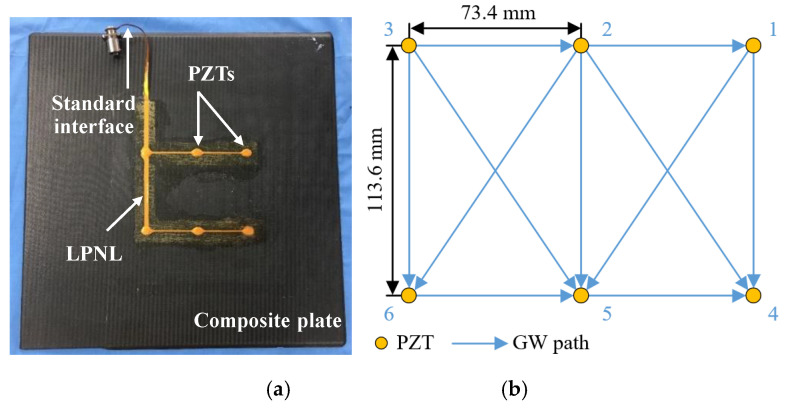
The adopted LPNL and its placement. (**a**)The surface mounted LPNL, (**b**) Placement of the PZT network.

Figure 3 shows the adopted LPNL. It has the dimensions of 358 mm × 163 mm and contains 6 PZTs numbered from 1 to 6. These PZTs form a network with 11 pitch–catch GW paths. A standard interface is used to input/output the GW signals of the whole network. The size of the composite plate is 460 mm × 460 mm × 3 mm (Length × Width × Thickness). For each above-mentioned environmental test, GW signals are acquired by an integrated SHM system [34]. The excitation signal adopted in this assessment is a five-cycle sine burst modulated by the Hanning window. A frequency sweep from 50 to 350 kHz with a frequency step of 10 kHz is performed at every test. The sampling rate is 10 MS/s, and the sampling length is 5000 sample points, including 500 pre-trigger sample points.

### 3.1. Temperature Tests

**Figure 4:**
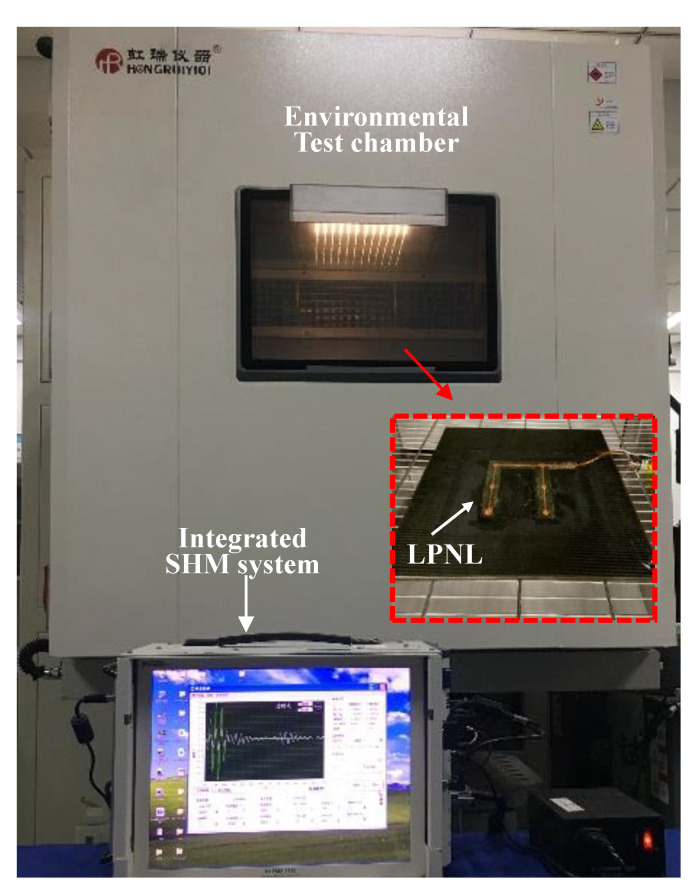
Experimental setup of temperature test.

The temperature tests aim at verifying the reliability and durability of the LPNL in harsh temperature environment. Figure 4 gives out the temperature test setup, an environmental test chamber THV1070W is used to place the LPNL and to provide the required temperature environment. The integrated SHM system is used to control the LPNL to actuate and acquire GW signals through a heat resisting cable and the standard interface. The temperature tests are carried out as follows.

**Low Temperature:** The temperature is set to be −50 °C and lasts for 280 min.

**High Temperature:** The temperature is set to be 150 °C and lasts for 280 min.

**Figure 5:**
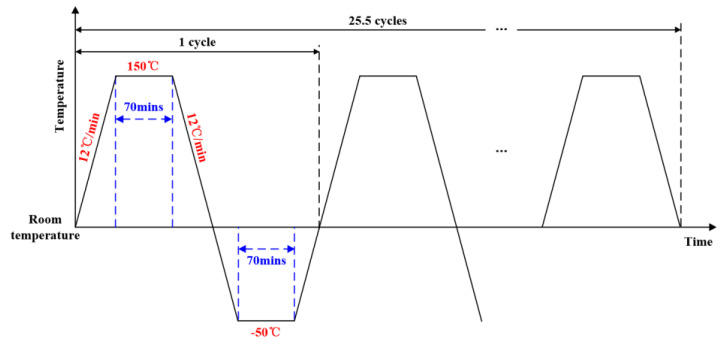
Implementation process of temperature variation.

**Thermal shock:** This is a dynamic temperature test, as shown in Figure 5. A total of 25.5 temperature cycles are carried out. For each cycle, the temperature varies from 150 °C to −50 °C at a high variation rate of 12 °C/min. This test lasts about 4420 min in total.

**Figure 6:**
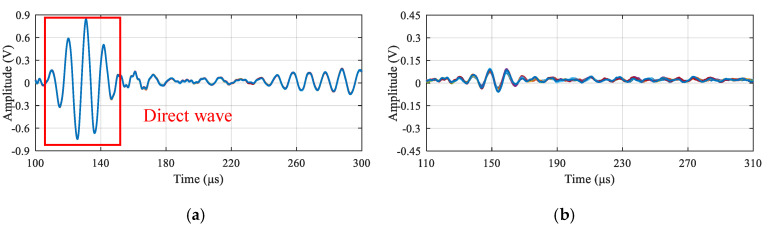
Acquired 8 GW signals of paths 2-6 at different temperatures. (**a**) Low temperature 50 °C. (**b**) High temperature 150 °C.

**Figure 7:**
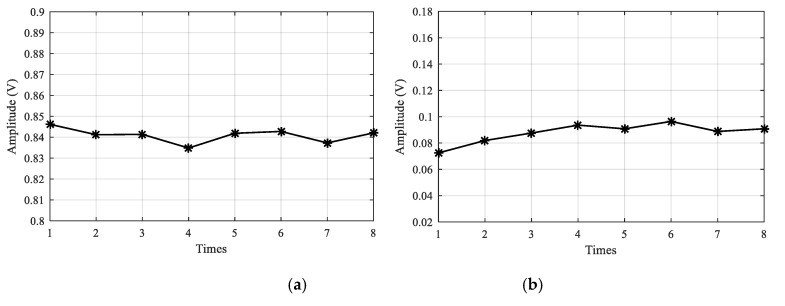
Amplitude plot of direct waves at different temperatures. (**a**) Low temperature –50 °C. (**b**) High temperature 150 °C.

For both the high temperature test and the low temperature test, GW signals of all the GW paths in the LPNL are acquired every 30 min to evaluate the signal stability. Taking path 2-6 as an example, Figure 6 shows its 8 GW signals acquired at −50 °C and 150 °C, respectively. Figure 7 gives out the corresponding amplitude plots of direct wave. It can be seen from this figure that the maximum amplitude difference of the 8 measurements is 0.011 V at −50 °C and 0.024 V at 150 °C, which means the LPNL can work stably under both low and high temperature.

**Figure 8:**
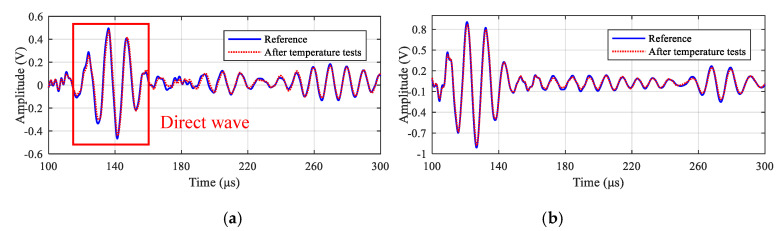
GW signals of different paths before and after temperature tests. (**a**) Path 2-6. (**b**) Path 3-6.

**Figure 9:**
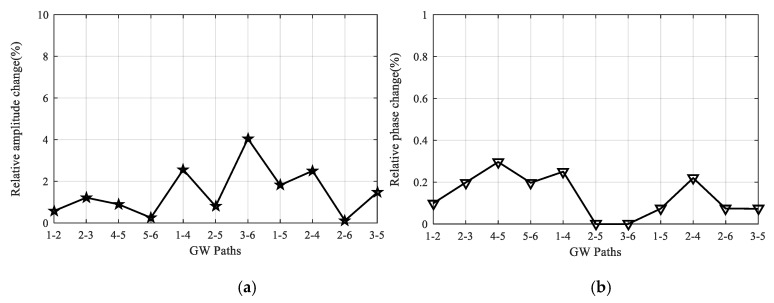
Relative signal change of all the paths after temperature tests. (**a**) Relative amplitude change. (**b**) Relative phase change.

After finishing all the temperature tests, GW signals of all the paths are acquired and compared with the reference signals obtained before the tests at room temperature. As an example, Figure 8 gives the comparison results of 90 kHz signals of paths 2-6 and 3-6, respectively. It can be seen that only minor changes occur for the signals of every path. Besides, the reliability under temperature tests is further assessed by calculating the relative change of signal amplitude and phase. For each path, assuming the maximum direct wave amplitude of its reference signal and the signal acquired after the test are *V_r_* and *V_a_*, respectively, then the relative change of signal amplitude *E_amp_* is given according to Equation (1). Similarly, the relative change of signal phase *E_pha_* can be calculated based on Equation (2). Figure 9 gives the *E_amp_* and *E_pha_* values of all the 11 paths and it can be seen that the maximum *E_amp_* value is less than 4.0% and the maximum *E_pha_* value is less than 0.3%, which indicates that the developed LPNL can withstand long-time harsh temperature environments and is still capable of working reliably.

(1) $$E_{amp}=\left|\frac{V_{r}-V_{a}}{V_{r}}\right|\times 100\%$$

(2) $$E_{pha}=\left|\frac{T_{r}-T_{a}}{T_{r}}\right|\times 100\%$$

### 3.2. Vibration Tests

**Figure 10:**
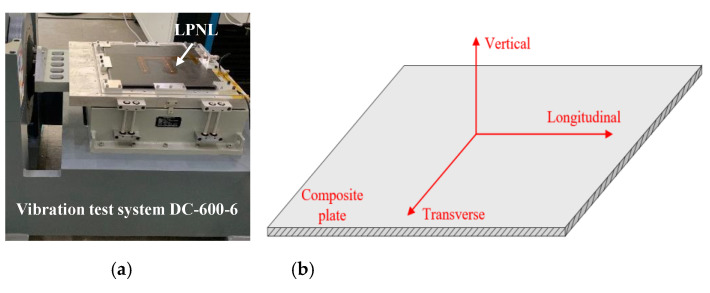
Experimental setup of vibration test. (**a**) Experimental setup. (**b**) Definition of vibration direction.

**Figure 11:**
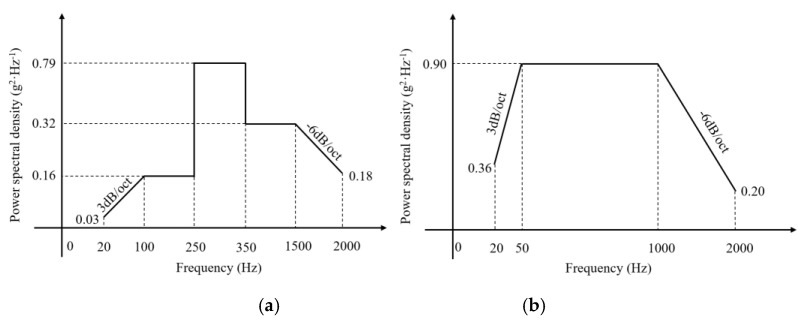
Profile of the two high frequency random vibrations. (**a**) High frequency random vibration 1. (**b**) High frequency random vibration 2.

According to the vibration test profile, two kinds of high frequency random vibrations corresponding to different flying phases are performed by adopting a vibration test system DC-600-6, as shown in Figure 10a. What should be noted is that, for each high frequency random vibration, vibration tests on longitudinal direction, vertical direction and transverse direction are carried out, respectively. Figure 10b shows the definition of directions. The first random vibration profile is shown in Figure 11a and described in Table 1. The frequency is from 20 Hz to 2000 Hz and the root mean square (RMS) acceleration is 24.3 g, which lasts for 7 min per direction. As for the second random vibration profile, it can be seen from Figure 11b and Table 2 that it has the same frequency range, while its RMS acceleration is as high as 36.4 g and lasts for 4.5 min per direction. GW signals of all the paths are acquired before and after each kind of vibration test.

**Figure 12:**
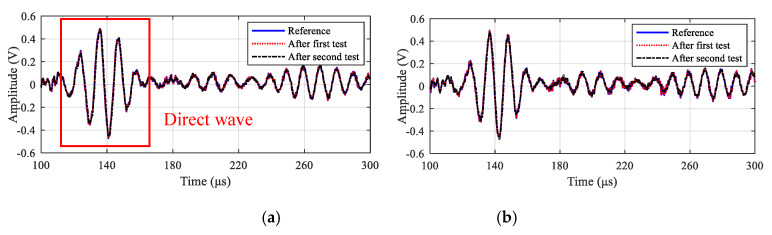
Signal comparison results of paths 2-6 and paths 3-5. (**a**) Paths 2-6. (**b**) Paths 3-5.

**Figure 13:**
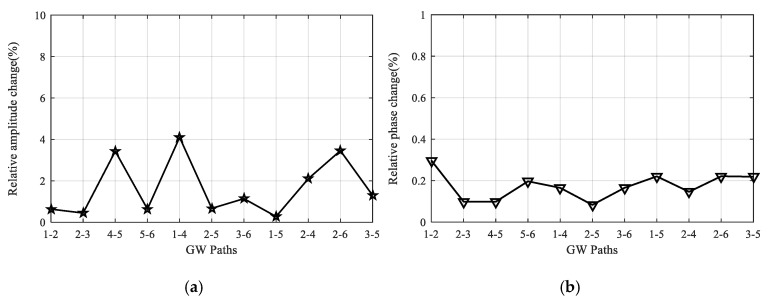
Relative signal change of all the paths after vibration tests. (**a**) Relative amplitude change. (**b**) Relative phase change.

In order to assess the integrity of LPNL under vibration test, the reference signals, signals acquired after every high frequency random vibration are compared with each other. Figure 12 gives the comparison results of path 2-6 and 3-5 at 90 kHz, respectively. It can be seen that, for each path, the signal basically remains unchanged after the two random vibration profiles. Besides, the GW signals of other frequencies and other paths are also compared, which show similar results with paths 2-6 and 3-5. The relative change in signal amplitude and phase of all the paths are also calculated. As shown in Figure 13, the maximum change is 4.1% and 0.3%, respectively. Therefore, the developed LPNL can withstand the two high frequency random vibration profiles and keep working normally, showing its high reliability in space applications.

### 3.3. Impact Tests

**Figure 14:**
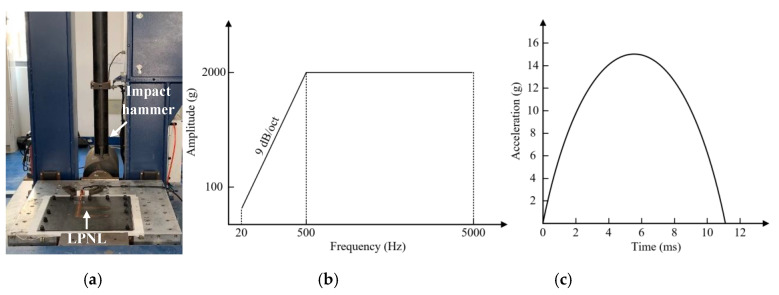
Experimental setup of impact test. (**a**) Experimental setup. (**b**) The impact response spectrum. (**c**) The half sinusoid spectrum.

Impact tests are carried out according to two different profiles, namely an impact-response spectrum and a half sinusoid spectrum. Figure 14a shows the experimental setup of the impact response spectrum-based test, the impact response spectrum testing machine SP80 is used to perform the profile in Figure 14b. The half sinusoid spectrum-based impact test is performed by adopting the vibration test system MPA3324/H1248A and the half sinusoid spectrum in Figure 14c. Like the vibration test, each impact profile is carried out on a longitudinal direction, vertical direction and transverse direction, respectively. Besides, what should be noted is that, for each direction mentioned above, both forward and backward impact tests are performed three times.

**Figure 15:**
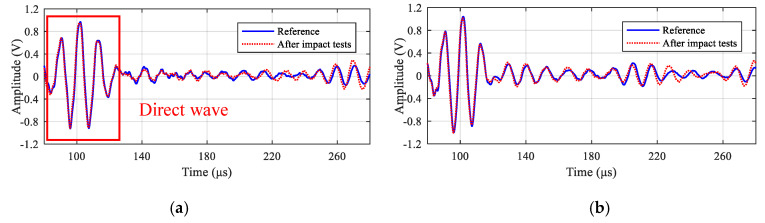
GW signals of different paths before and after impact tests. (**a**) Path 1-2. (**b**) Path 2-3.

**Figure 16:**
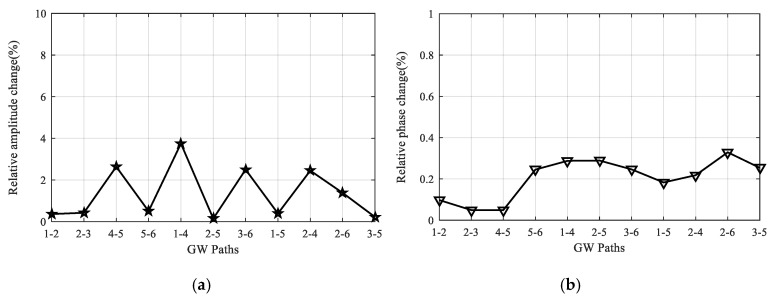
Relative signal change of all the paths after impact tests. (**a**) Relative amplitude change. (**b**) Relative phase change.

In total, the impact response spectrum and the half sinusoid spectrum are both performed 18 times. GW signals of all 11 paths in the LPNL are acquired after the two kinds of impact tests and are compared with reference signals obtained before the impact tests. Taking the 90 kHz signals of paths 1-2 and 2-3 as examples, Figure 15 shows their signal comparison results. It can be seen that the recorded signals are barely affected by the impact. Besides, Figure 16 gives the relative change in signal amplitude and phase of every path, respectively. The maximum relative change of signal amplitude of all the paths is 3.8%, and the maximum relative change of signal phase is 0.3%.

### 3.4. Overload Tests

**Figure 17:**
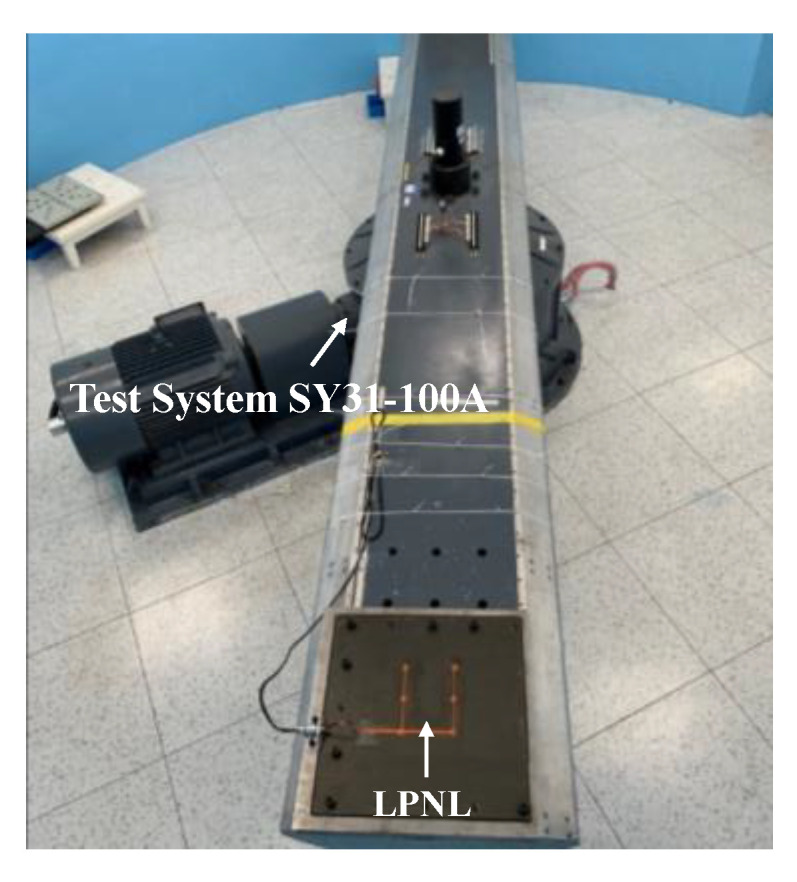
Experimental setup of overload tests.

An arm rotating constant acceleration testing system SY31-100A is adopted to provide the required acceleration of the overload tests. As shown in Figure 17, the composite plate with the LPNL is fixed on the testing system. The overload test is also included in three directions, as mentioned above. For each direction, both forward and backward overload are performed for 1 minute. According to the test profile given in Table 3, every direction has its corresponding acceleration values. GW signals are acquired before and after the entire overload tests.

**Figure 18:**
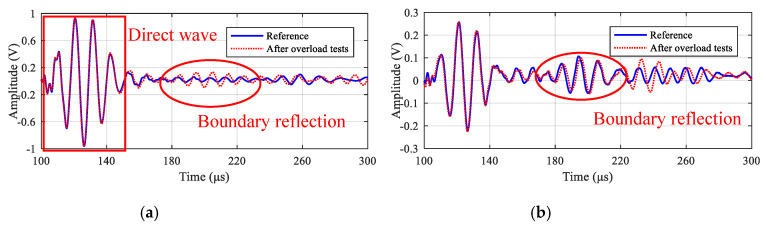
Signal comparison results of path 2-5 and 1-4 before and after overload tests. (**a**) Path 2-5. (**b**) Path 1-4.

**Figure 19:**
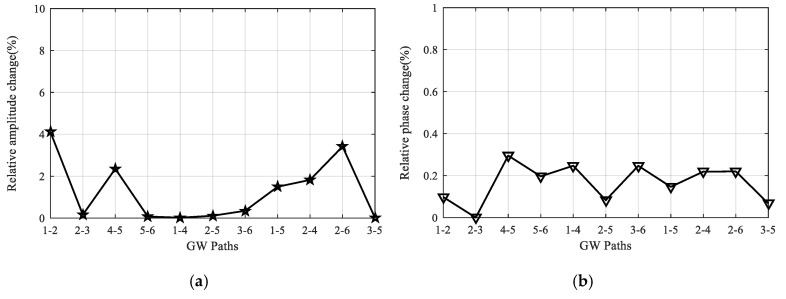
Relative signal change of all the paths after overload tests. (**a**) Relative amplitude change. (**b**) Relative phase change.

Taking paths 2-5 and 1-4 as examples, Figure 18 gives their signal comparison results before and after the overload test. It should be noted that the direct wave shows minor changes, while the boundary reflections have relatively large change, which could be caused by the change in boundary conditions after the overload tests. Additionally, the reliability under overload tests is further assessed by calculating the relative change in signal amplitude and phase of every path. Figure 19 gives the relative amplitude and phase change of all the 11 paths, and it can be seen that the maximum values are about 4.1% and 0.3%, respectively, which shows the reliability of the LPNL.

### 3.5. Damage Diagnostic Application

After finishing all the reliability tests mentioned above, a damage diagnosis experiment is further performed to verify the LPNL’s SHM functionality. A kind of solid adhesive tape, which is capable of interrupting the structural geometry continuum, just as the effect of real damage, is adopted as simulated damage [35]. In this work, four simulated damages numbered from damages 1 to 4 are bonded on different places of the composite plate. The dimension of the simulated damage is 10 mm × 10 mm, and the thickness is 3 mm.

**Figure 20:**
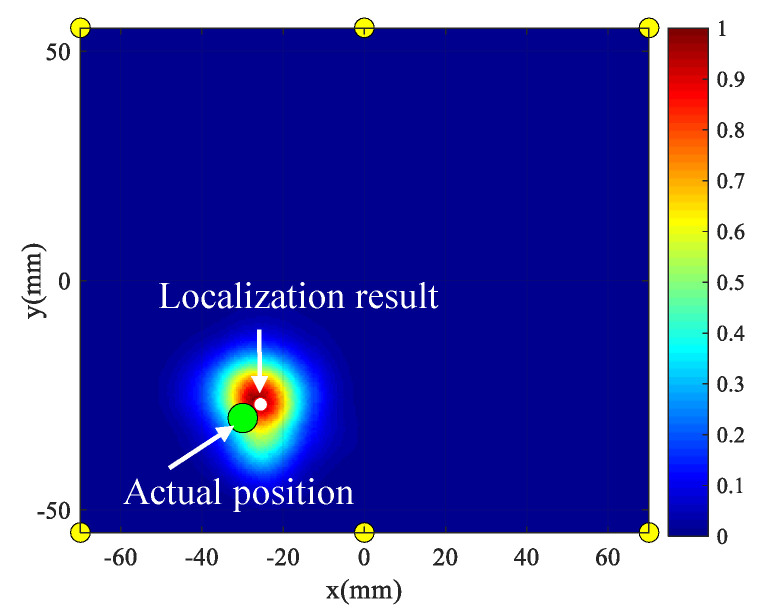
Imaging result of Damage 1.

Take damage 1 as an example, the damage scattering signals of all the paths are first extracted, which show the capability of generating detectable wave scatter from damage. Based on these scattering signals, the conventional delay-and-sum imaging method [36] is adopted to locate damage. Figure 20 shows the damage imaging and localization results of damage 1—The green circle represents the actual damage position and the white circle represents the localization result. It can be seen that the localization result is in good agreement with the actual damage position. The maximum localization error of all the four damages is 0.41 cm, which indicates that the LPNL can still perform well after the extreme space environment tests.

## 4. Conclusions

Aiming at PZT and GW-based onboard SHM applications of space structures, this paper proposes a design method of large-scale PZT network layers. By adopting the FPC technology, the method first realizes the lightweight design of a large-scale PZT network without additional weight caused by traditional cables. Then, a network splitting–recombination method is proposed to overcome the current manufacturing limitation on the network’s dimension and obtain the reliable integration of LPNL. The developed LPNL has the advantages of being large size, lightweight, ultra-thin, flexible, customized in shape and high reliable. Additionally, a series of extreme environmental conditions tests, including temperature, vibration at different flying phases, landing impact, and flying overload, are performed to verify the reliability of the developed LPNL.

However, considering the service environment of space structures, the reliability assessment of LPNL that has been carried out is not sufficient enough. In the ongoing research, the performance of the developed LPNL under other extreme space conditions, such as radiation and vacuum will be studied. The proposed design method will be further improved according to the assessment results.

## Figures and Tables

**Table 1:** High frequency random vibration condition 1.

Frequency (Hz)	Power Spectra Density (g^2^/Hz)	RMS Acceleration (g)	Duration
20~100	3 dB/oct	24.3	7 min/direction
100~250	0.16		
250~350	0.79		
350~1500	0.32		
1500~2000	−6 dB/oct		

**Table 2:** High frequency random vibration condition 2.

Frequency (Hz)	Power Spectra Density (g^2^/Hz)	RMS Acceleration (g)	Duration
20~50	3 dB/oct	36.34	4.5 min/direction
50~1000	0.9		
1000~2000	−6 dB/oct		

**Table 3:** Overload test conditions.

Direction		Overload (g)	Duration Time
Longitudinal acceleration	Forward	6	1 min
	Backward	2	1 min
Vertical acceleration	Forward	4.5	1 min
	Backward	4.5	1 min
Transverse acceleration	Forward	2	1 min
	Backward	2	1 min
